# Assessing quality of life on the day of chemotherapy administration underestimates patients’ true symptom burden

**DOI:** 10.1186/1471-2407-14-758

**Published:** 2014-10-10

**Authors:** Johannes M Giesinger, Lisa M Wintner, August Zabernigg, Eva-Maria Gamper, Anne S Oberguggenberger, Monika J Sztankay, Georg Kemmler, Bernhard Holzner

**Affiliations:** Department of Psychosocial Research and Epidemiology, The Netherlands Cancer Institute, Pesmanlaan 121, 1066 CX Amsterdam, The Netherlands; Department of Psychiatry and Psychotherapy, Innsbruck Medical University, Anichstr.35, A-6020 Innsbruck, Austria; Leopold-Franzens-University of Innsbruck, Innrain 52, A-6020 Innsbruck, Austria; Department of Internal Medicine, Kufstein County Hospital, Endach 27, A-6330 Kufstein, Austria

## Abstract

**Background:**

In chemotherapy trials quality of life (QOL) is assessed mostly at the days of chemotherapy administration (i.e. event-driven) during treatment and follows fixed time intervals in the aftercare phase (i.e. time-driven). Specific QOL impairments and treatment side-effects are known to be time dependent following different trajectories. Therefore, acute problems are likely to be missed if assessments are done infrequently or at inappropriate time points. Since the planning of supportive care interventions during chemotherapy depends on knowledge about symptom trajectories, such information may be of substantial importance to a clinician.

**Methods:**

Cancer patients receiving chemotherapy at Kufstein County Hospital were assessed using an electronic version of the EORTC QLQ-C30 at the day of chemotherapy administration at the hospital. One and two weeks later assessments were repeated via the internet while patients were at home. Assessments at home and the hospital were conducted using the web-based software CHES. Data were analysed by means of linear mixed models.

**Results:**

A sample of 54 chemotherapy outpatients participated in electronic QOL assessments at the hospital and at home. For 9 out of the 15 QOL domains of the EORTC QLQ-C30 patients reported increased burden one week after chemotherapy administration compared to the day of chemotherapy administration. Most pronounced differences were found for Fatigue, Constipation, and Appetite Loss.

**Conclusions:**

Our results indicate that patients experience most severe QOL impairments in the week following chemotherapy administration. This is a period that is usually not covered by QOL assessments in chemotherapy trials which may result in underestimation of true treatment burden. Our findings suggest to conduct QOL assessments not only event- or time-driven, but to rely on specific hypotheses on symptom and functioning trajectories.

## Background

The value of the information gained from patient-reported outcome (PRO) assessments in clinical trials and daily clinical practice relies substantially on the use of valid and reliable measurement instruments, being appropriate for the targeted patient group, and on the correct timing of the PRO assessment [1]. Whereas investigation of psychometric properties of PRO instruments and their applicability to specific patient groups has been a major research focus, the timing of the assessment has gained less attention. In clinical trials and in daily clinical practice PRO assessment is either event-driven (e.g. at every hospital visit) or time-driven (e.g. every three months) [2]. Such straightforward schedules allow simplifying data collection logistics, but may also have an impact on the collected PRO scores as symptom burden, functioning, and quality of life (QOL) may not be assessed at time points giving the most appropriate picture of their longitudinal development.

To date, only few studies have looked on the impact of timing of QOL assessments on QOL results. Retrospective analyses [3–5] of trial data compared assessments at the day of chemotherapy (CT) administration with deviating assessments that were collected outside the predefined time windows. These analyses, considering early or late assessments as a possible source of measurement error, found that for various QOL domains the scores were worse when collected one week after CT administration compared with later assessments.

According to the clinical model for QOL assessments suggested by Klee et al. [6] timing of QOL assessments should not just rely on fixed time patterns or clinical events, but follow considerations on the hypothesized course of a symptom or functioning domain. These authors differentiate between cancer-related symptoms and symptoms due to other causes, as well as between acute and chronic treatment side effects.

Defining adequate assessment time points for PROs appears to be most important for acute side effects, such as fatigue, nausea/vomiting, diarrhoea and appetite loss, since due to their limited duration they may be missed if assessment intervals are too large. Therefore the common assessment pattern in CT trials administering questionnaires only at the day of CT administration is likely to miss time points of high levels of acute side-effects. This is probably especially true, if CT protocols rely on three-weekly administration of cytostatic drugs. This has been shown in a study by Hilarius et al. [7] investigating acute and delayed chemotherapy-induced nausea and vomiting. The authors found prevalence rates for both symptoms to be nearly twice as high a week after CT administration when compared to the day prior to CT administration.

Inadequate QOL assessment schedules in clinical trials may result in biased QOL underestimating the true symptom burden a patient experiences. In comparative trials differences between chemotherapy regimens with regard to acute side effects may go unnoticed in case of infrequent QOL assessments and therefore lead to incorrect evaluation of the impact of treatments on patient’s QOL. In daily clinical practice QOL assessments covering the period following CT administration may allow timely interventions to manage patients symptom burden at the moment of highest severity.

Based on such considerations we aim at investigating if symptom burden and functioning impairments recorded at the time of CT administration differ substantially from assessments one and two weeks later, thus requiring more frequent assessment schedules in future trials or when monitoring symptoms in daily clinical practice. For this purpose our study made use of web-technology to extend PRO assessments beyond the hospital setting. In detail, the study compares CT patients’ QOL at three time points: at the day of CT (at the hospital) administration, one week later and two weeks later (both at home).

## Methods

### Sample

In this prospective study we recruited cancer patients undergoing CT at the Department of Internal Medicine at Kufstein County Hospital (Austria) according to the following inclusion criteria:

-  any cancer diagnosis
-  CT regimen with 2-weekly or 3-weekly administration of cytostatic drug
-  age above 18
-  no obvious cognitive impairments
-  no language barriers
-  written informed consent

Socio-demographic and clinical patient characteristics were gathered from the medical charts. Patients were included at administration of any CT cycle and then assessed during multiple cycles. We conducted one assessment between cycles if patients received a two-weekly regimen and two if patients received a three-weekly regimen.

The study has been approved by the Ethics Committee of Innsbruck Medical University.

### QOL data capture

Patients’ QOL was assessed with the EORTC QLQ-C30 [8], an internationally validated and widely used questionnaire to assess QOL, psychosocial burden and physical symptoms in cancer patients. It consists of five functioning scales (Physical, Social, Role, Cognitive, and Emotional Functioning), a scale for Global QOL, and nine symptom scales (Fatigue, Pain, Nausea/Vomiting, Dyspnoea, Appetite Loss, Sleep Disturbance, Constipation, Diarrhoea and Financial Difficulties). The QLQ-C30 uses a recall period of one week which provided the rationale for our weekly assessment schedule. This means that for example an assessment one week after chemotherapy administration provides information on the QOL experienced in the week following chemotherapy, whereas an assessment at the day of chemotherapy administration provides information on pre-administration QOL.

After study inclusion patients completed their first QOL assessment with the QLQ-C30 in the hospital on a tablet PC supervised by a study nurse who answered questions concerning the use of the electronic device. Patients were then offered to complete the follow-up questionnaires one and two weeks later at home either via the web-browser on their own computer, or via a tablet PC that they could obtain from the hospital for the duration of study participation. In both cases the patients received an information sheet explaining how to log on to the system from home and a user name and password to do so.

Electronic questionnaire administration at the hospital and at home was done with the Computer-based Health Evaluation System (CHES) [9, 10], a software for PRO data collection and result presentation in daily clinical practice and clinical trials.

### Statistical analysis

Analysis of QOL differences between hospital- and home-based assessments was done with linear mixed models. The model comprised assessment time point within CT cycle (at hospital, 1 week after CT at home, and 2 weeks after CT at home), time since diagnosis and the interaction term as fixed effects. In addition, it employed a 1st order autoregressive covariance structure and a random intercept at patient level. In this model the two-way interaction term for time point and time since diagnosis indicates that the size of the differences between the three time points (at hospital, 1 week and 2 weeks later) changes over the course of treatment.

The main analysis used the time window of 4–10 days after hospital-based assessment for the one-week time point, and 11–17 days for the two-weeks time point. We would like to note that in our statistical model the QOL data collected from the same patient during different CT cycles was summarised time point-wise. This means for example that for a patient receiving a two-weekly regimen, assessments from different cycles were either classified as "at hospital" or as "one-week follow-up" and then compared against each other.

To investigate robustness of our results we performed additional sensitivity analyses using different time windows for the follow-up time points.

According to Osoba et al. [11] a difference of at least 5 points is considered a small clinical difference on the QLQ-C30 scales, whereas a difference of at least 10 points reflect a moderate clinical difference. This is in line with the thresholds for clinical relevance suggested by Ringash et al. [12]. As additional indicators of differences in symptom burden at home and in the hospital we calculated percentages of patients for which hospital-reported symptom burden is at least 10 points (i.e. moderately) worse than symptom burden reported at home.

## Results

### Patient characteristics

One hundred sixty-six patients were eligible for study participation, of which 55 patients (33.1%) were willing to complete questionnaires electronically (including web-based assessments at home). One of the 55 patients completing questionnaires web-based at home did not provide corresponding assessments at the hospital and was therefore excluded from analysis. Thirty-four patients obtained a tablet PC from the hospital for questionnaire completion at home and 20 patients used their own computer to access the questionnaires online.

One hundred and eleven patients did not want to complete questionnaires electronically at home (51 preferred being assessed at home via phone interviews, 28 were skeptic about QOL assessments and electronic assessments in general, 15 wanted to be assessed only at the hospital, 6 were in a poor physical condition, and 11 refused for other reasons). Details on the feasibility of our web-based assessments are published elsewhere.

Patients participating in phone interviews were excluded from analysis as mode of administration (interview vs. electronic questionnaire) would otherwise be a source of bias [13–15]. Participants and non-participants did not differ significantly with regard to sex (55.6% vs. 52.3% male; p = 0.741), but with regard to age (58.7 vs. 68.7 years; p < 0.001).

Mean age of the 54 participants was 58.7 years (SD 10.8) and 55.6% were men. Most frequent diagnoses were lung cancer (25.9%) and breast cancer (20.4%). Total number of assessments was 561, i.e. patients completed the QLQ-C30 10.4 times on average. 41.1% of the assessments were conducted electronically at the hospital at the day of CT administration, 36.1% one week (4–10 days) later, and 22.7% two weeks (11–17 days) later at home via internet.

Most patients (68.8%) were receiving CT with palliative intent, with taxane monotherapy (15.9%) and gemcitabine monotherapy (10.5%) being the most frequent regimens. For further details see Table 1.

**Table 1:** **Descriptive statistics for sociodemographic and clinical variables (n = 54)**

**Age:**	Mean (SD)	58.7 (10.8)
	Median	61
**Sex: n (%)**	Men	30 (55.6%)
	Women	24 (44.4%)
**Time since diagnosis (weeks):**	Mean (SD)	52.5 (70.5)
	Median	19
**Diagnosis: n (%)**	Lung cancer	14 (25.9%)
	Breast cancer	11 (20.4%)
	Lymphoma	8 (14.8%)
	Pancreatic or cholangiocellular cancer	8 (14.8%)
	Colorectal cancer	7 (13.0%)
	Ovarian cancer	4 (7.4%)
	Stomach cancer	1 (1.9%)
	Oesophagic cancer	1 (1.9%)
Chemotherapy: n (%)*	Taxane monotherapy	89 (15.9%)
	Gemcitabine	59 (10.5%)
	Capecitabine plus platin	33 (5.9%)
	Irinotecan and capecitabine	33 (5.9%)
	Ribomustin	30 (5.3%)
	Platin plus etoposid	28 (5.0%)
	Taxane plus Bevacizumab	25 (4.5%)
	Taxane plus platin	25 (4.5%)
	Irinotecan, capecitabine and bevacizumab	22 (3.9%)
	Pemetrexed plus platin	19 (3.4%)
	Vinorelbin plus platin	19 (3.4%)
	Irinotecan	17 (3.0%)
	Other	28.9%
Chemotherapy line: n (%)*	Neoadjuvant	58 (10.3%)
	Adjuvant	91 (16.2%)
	Curative	26 (4.6%)
	1. Line palliative	183 (32.6%)
	2. Line palliative	111 (19.8%)
	3. Line or more palliative	92 (16.4%)

*percentages refer to total number of assessments (n = 561).

### Comparison of QOL assessments at the hospital and at home

The comparison of the QOL assessments at the hospital and one and two weeks later at home showed a consistent pattern across many functioning and symptom scales of the QLQ-C30. The highest symptom burden was usually observed one week after CT administration and partial or complete recovery after two weeks to those levels reported at the hospital visit.

**Figure 1:**
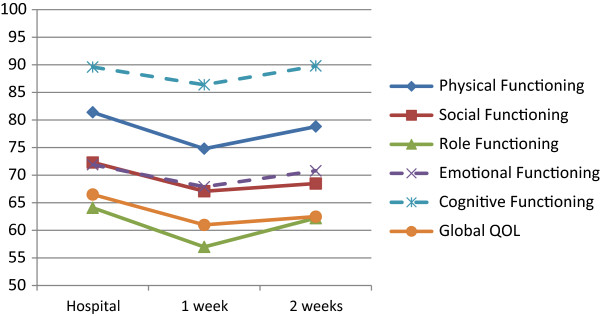
**EORTC QLQ-C30 functioning scores at the hospital and 1 and 2 week(s) later at home.** Dashed lines indicate that differences between hospital- and home-based assessments do not reach clinical relevance (i.e. a 5 point difference [11]).

**Figure 2:**
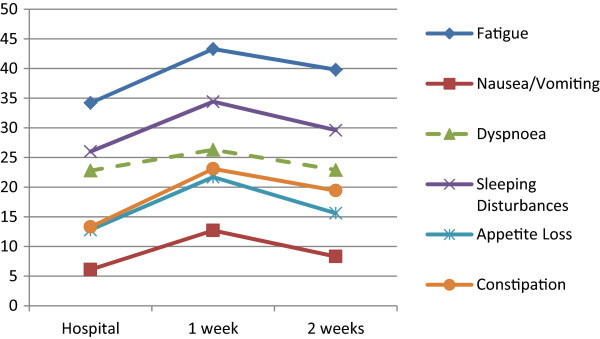
**EORTC QLQ-C30 symptom scores at the hospital and 1 and 2 week(s) later at home.** Dashed lines indicate that differences between hospital- and home-based assessments do not reach clinical relevance (i.e. a 5 point difference [11]); non-significant scores not shown.

Assuming a five-point-difference as threshold for clinical relevance [11] the following scales showed clinically relevant (and statistically significant) mean differences between the hospital-based assessment and the follow-up one week later at home:

Constipation (9.8 points), Fatigue (9.1 points), Appetite Loss (8.9 points), Sleeping Disturbances (8.4 points), Role Functioning (-7.1 points), Physical Functioning (-6.6 points), Nausea/Vomiting (6.6 points), Global QOL (-5.5 points), and Social Functioning (-5.2 points). All differences indicated higher impairment at home one week after CT administration compared to the assessment at the hospital. Further details are given in Tables 2 and 3 as well as in Figures 1 and 2.

**Table 2:** **EORTC QLQ-C30 functioning domains and time point of questionnaire completion (54 patients, 561 QOL assessments)**

EORTC QLQ-C30	At the hospital	At home (1 week later)	At home (2 weeks later)	Time point (main effect) F/p values
Estimated mean (95% CI)				
Physical Functioning	81.4 ^1w^ (76.9-86.0)	**74.8** ^**Ho, 2w**^ **(70.2-79.4)**	78.8 ^1w^(74.1-83.6)	F = 13.32; p < 0.001
Social Functioning	72.3 ^1w, 2w^(66.5-78.0)	**67.1** ^**Ho**^ **(61.3-72.9)**	68.5 ^**Ho**^(62.5-74.6)	F = 6.22; p = 0.002
Role Functioning	64.1 ^1w^(58.2-70.0)	**57.0** ^**Ho, 2w**^ **(51.1-63.0)**	62.2 ^1w^(55.9-68.4)	F = 11.16; p < 0.001
Emotional Functioning	71.9 ^1w^(67.2-72.6)	67.9 ^Ho, 2w^(63.1-72.6)	70.8 ^1w^(65.9-75.7)	F = 6.66; p = 0.001
Cognitive Functioning	89.6 ^1w^(85.6-96.5)	86.4 ^**Ho, 2w**^(82.4-90.3)	89.8 ^1w^(85.6-93.9)	F = 6.91; p = 0.001
Global QOL	66.5 ^1w, 2w^(62.6-70.5)	**61.0** ^**Ho**^ **(57.0-65.0)**	62.5 ^Ho^(58.3-66.7)	F = 12.1; p < 0.001

superscript numbers indicate significance (p < 0.05) of post-hoc group comparisons (LSD-test).

score differences to the hospital-based assessment exceeding 5 points are given in bold letters.

**Table 3:** **EORTC QLQ-C30 symptom domains and time point of questionnaire completion (54 patients, 561 QOL assessments)**

EORTC QLQ-C30	At the hospital	At home (1 week later)	At home (2 weeks later)	Time point (main effect) F/p values
Estimated mean (95% CI)				
Fatigue	34.2 ^1w, 2w^(29.2-39.3)	**43.3** ^**Ho, 2w**^ **(38.2-48.3)**	**39.8** ^**Ho, 1w**^ **(34.4-45.1)**	F = 21.40; p < 0.001
Nausea/Vomiting	6.1 ^1w^(3.3-8.9)	**12.7** ^**Ho, 2w**^ **(9.8-15.5)**	8.3 ^1w^(5.2-11.4)	F = 19.89; p < 0.001
Pain	18.7(13.5-24.0)	21.7(16.4-27.0)	21.6(16.0-27.1)	F = 2.51; p = 0.083
Dyspnoea	22.8 ^1w^(16.1-29.5)	26.3 ^**Ho, 2w**^(19.6-33.1)	22.9 ^1w^(16.0-29.8)	F = 3.92; p = 0.021
Sleeping Disturbances	26.0 ^1w^(20.1-32.0)	**34.4** ^**Ho, 2w**^ **(28.4-40.5)**	29.6 ^1w^(23.3-36.0)	F = 12.37; p < 0.001
Appetite Loss	12.8 ^1w^(7.5-18.1)	**21.7** ^**Ho, 2w**^ **(16.4-27.1)**	15.6 ^1w^(9.9-21.3)	F = 14.94; p < 0.001
Constipation	13.3 ^1w, 2w^(8.1-18.5)	**23.1** ^**Ho**^ **(17.8-28.4)**	**19.4** ^**Ho**^ **(13.7-25.2)**	F = 13.70; p < 0.001
Diarrhoea	7.1(4.1-10.2)	7.3(4.2-10.3)	6.0(2.6-9.3)	F = 0.46; p = 0.634
Financial Impact	16.6(10.5-22.7)	18.4(12.3-24.5)	18.9 (12.6-25.1)	F = 2.94; p = 0.054

superscript numbers indicate significance (p < 0.05) of post-hoc group comparisons (LSD-test).

score differences to the hospital-based assessment exceeding 5 points are given in bold letters.

We did not find a significant interaction of time since diagnosis and assessment time point for any of the QLQ-C30 scales, i.e. the observed differences between the hospital-based assessment and the two assessments at home did not change significantly over the course of treatment.

With regard to the proportion of assessments showing at least a 10 point worse score at the one-week follow-up at home compared to the hospital-based assessment we found the by far highest frequency for Fatigue (50.3%), followed by Role Functioning (33.0%), Sleeping Disturbances (32.3%), Nausea/Vomiting (30.9%), and Global Quality of Life (30.0%). The smallest proportions were observed for Diarrhoea (6.8%) and Financial Impact (10.5%). All other scales showed frequencies between 15% and 30%.

### Sensitivity analysis

Further analysis was done to investigate how robust our results are against changes in the definition of the time windows for the individual time points.

Narrowing down the time window from 7 and 14 days plus/minus 3 days as in the main analysis to plus/minus 2 days reduced number of assessments included in the analysis from 561 to 480. For this time point definition the estimated means differed from the main analyses only slightly. For seven of the 45 comparisons (15 scales - three time points) differences to the main analysis were between 1.0 and 1.9 points whereas all other deviations were below 1.0 point. Further restriction of the time window to plus/minus 1 day allowed 426 assessments to be analyzed. Deviations from the original means were between 1.0 and 2.0 points for 11 comparisons and 2.4 and 2.9 points for two comparisons. As these deviations were well below thresholds for clinical relevance we consider these results as indicating sufficient robustness of the main results.

For further exploratory analysis we split the time variable in five smaller categories (0, 4–7, 8–11, 12–15 and 16–17 days after CT administration) to identify the peak in QOL impairments in an exploratory way to guide future studies. As five categories come with 10 pairwise comparisons we consider this analysis exploratory due to effects of conducting multiple tests. The analysis indicated that the highest QOL impairments occurred between day 4 and 7 after CT for Physical Functioning, Social Functioning, Role Functioning, Fatigue, Appetite Loss, and Constipation. Between day 8 and 11 the following scales showed highest impairments: Cognitive Functioning, Global QOL, Nausea/Vomiting, and Financial Impact. For Emotional Functioning, Pain, Dyspnoea, Sleep Disturbances and Diarrhoea score differences between these two periods (i.e. 4–7 and 8–11 days) were below 1 point and higher levels were higher than at the other three periods (i.e. at hospital, or 12–15 and 16–17 days).

## Discussion

Our study showed that in CT outpatients patient-reported symptom burden and functioning impairments are most severe one week after CT administration. According to the one-week time frame of the QLQ-C30 this assessment covers those days immediately following CT administration. For 9 out of the 15 QOL domains covered by the QLQ-C30 the differences between the assessment at the day of CT and one week later exceeded the threshold for a minimal important clinical change [11], with strongest increase in burden found for Fatigue, Constipation, and Appetite Loss. Two weeks after administration impairment levels mostly recovered to those assessed at the time of the outpatient hospital visit. These results indicate that assessments taking place at the hospital at the time of CT administration underestimate true symptom burden experienced by the patient in the following week, i.e. when s/he is usually at home.

Due to the heterogeneity of the sample our analysis did not allow detailed conclusions for specific CT regimens. However, one would expect that heterogeneity of diagnosis and treatment results in blurred symptom trajectories not showing a clear pattern of delayed side effects one week after treatment that decrease before the next hospital visit. Given that our data still allowed clearly identifying such a pattern, this effect may be rather uniform across patient groups and might be considerably stronger for specific treatments. Besides, our sample was comparably young for cancer population (mean age 59 years), since completing the PRO measures on the internet requires a certain degree of computer-literacy. Albeit, provision of tablet PCs to patients helped to include also patients not being familiar with computers, using them only infrequently or not at all before study participation. Although this affects the validity of the absolute scores, i.e. the means at the various time points, the mean differences are probably more robust. For example, the for a cancer population rather low mean age of 59 years probably gives a biased estimate of the Physical Functioning means, whereas the effect, that Physical Functioning is lowest one week after CT administration and then recovers, is probably also valid for older cancer patients.

A strength of our study is that we had the same mode of administration (electronic) in the hospital and at home, so we could rule out a mode of administration effect that would have been introduced by collecting PRO data e.g. on a tablet PC at the hospital and via phone interview at home. Statistical power of the main analysis was sufficient as indicated by statistically significant differences that are below the threshold for clinical relevance.

We consider our findings to be well in line with clinicians’ expectations concerning trajectories of acute side effects and with the literature. The analysis of Pater et al. [4] on the optimal timing of completion of the QLQ-C30 when assessing nausea and vomiting in antiemetic trials found that accurate assessment of quality of life should be based on an assessment time point and a recall period covering the first three days after CT administration (e.g. a seven day recall period with an assessment at day 8 after chemotherapy administration). Inadequate timing led to clinically relevant differences of clinical trial results. As in our study QOL deteriorated during the first week following chemotherapy administration. Hakamies-Blomqvist et al. [5] showed that in a trial comparing docetaxel and methotrexate combined with 5-fluorouracil, timing of assessment had an impact on patients’ reports of e.g. Global QOL, Physical Functioning, Fatigue, and Nausea. Whereas that study only compared correctly (i.e. at the day of treatment or up to four days earlier) against incorrectly timed assessments, a more recent analysis of three EORTC trials on CTs done by Ediebah et al. [3] compared early, accurate, and late PRO assessments. The authors found that QOL assessments with the QLQ-C30 at the day of CT administration did not differ from those up to 10 days prior to chemotherapy administration. However, similar to our study QOL assessments made up to ten days after CT administration showed stronger impairments. In all three trials Nausea/Vomiting was worse when assessed up to 10 days after administration (3–6 points), and in two of three trials this was also observed for Fatigue (4–8 points), Appetite Loss (5–7 points) and Social Functioning (4–6 points).

Such findings have several implications for conducting PRO assessments. First, they underline the importance of following a strict schedule when assessing PRO in a clinical trial, as deviations may alter results. Second, these studies demonstrate the importance of incorporating specific hypotheses on the course of PRO domains in a trial design. Third, PRO assessments at the day of treatment administration may in general underestimate true symptom burden as side effects develop after the assessment and may disappear before the subsequent assessment.

Naturally, as patients often attend the hospital only for treatment administration and spend time between treatments at home, more adequate timing of PRO assessments requires patients to complete questionnaires outside the hospital. An obvious, while not the only, method of collecting data while patients are not in the hospital is the use of web-sites where patients can log on and complete PRO instruments. The feasibility of this method for data collection has been demonstrated in various cancer patient populations [16–18]. Despite an increase in computer and internet literacy in elderly populations [19], the implementation of a routine web-based PRO assessment still remains challenging.

## Conclusion

In conclusion, we think that our findings should encourage researchers and clinicians to schedule PRO assessments not only in accordance with clinical events or fixed follow-up time points, but also to rely on considerations on hypothesised symptom trajectories. In chemotherapy patients QOL assessments only at the day of CT administration underestimate the true symptom burden. Based on our results and the literature we recommend to assess QOL also one week after CT administration when patients are at home. This may help to obtain a more complete picture of the burden and impairments a patient is confronted with. The comprehensive understanding of patients’ symptom burden is crucial for treatment optimization and individualisation, as such knowledge may help to optimize supportive care during chemotherapy.
